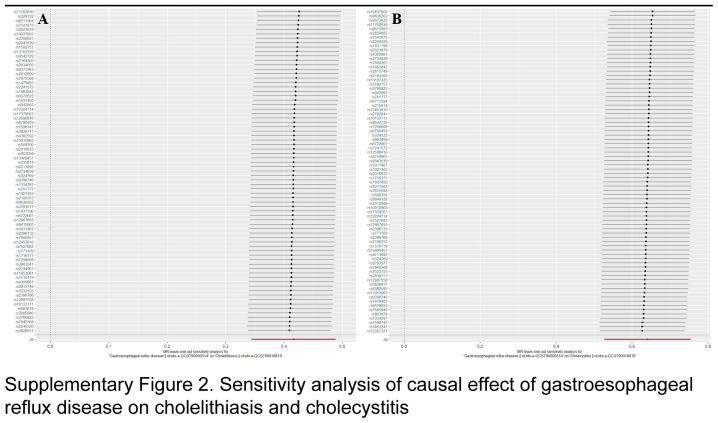# Exploring the causal relationships between cholelithiasis, cholecystitis, cholecystectomy, and gastroesophageal reflux disease: a bidirectional two-sample Mendelian randomization study

**DOI:** 10.1097/JS9.0000000000001992

**Published:** 2024-08-02

**Authors:** Huahang Lin, Runda Lu, Qixin Shang, Yimin Gu, Yixin Liu, Yushang Yang, Longqi Chen

**Affiliations:** Department of Thoracic Surgery, West China Hospital of Sichuan University, Chengdu, Sichuan Province, People’s Republic of China

**Keywords:** cholelithiasis, cholecystitis, cholecystectomy, gastroesophageal reflux disease, Mendelian randomization

## Abstract

**Background:**

Biliary disorders and gastroesophageal reflux disease (GERD) frequently coexist. However, precise linkages between these conditions remain to be clarified.

**Methods:**

Univariable Mendelian randomization (MR), Bayesian weighted MR (BWMR) along with multivariable MR approaches were conducted using genetic instruments to evaluate the causality involving biliary disorders and GERD. Furthermore, an investigation was conducted on the potential mediating roles of biliary disorders (or GERD), on the linkage involving BMI and GERD (or biliary disorders).

**Results:**

Univariable MR analyses revealed significant causal effects of genetically predicted cholelithiasis [odds ratio (OR)=1.04, *P*=0.0001], cholecystitis (OR=1.06, *P*=0.0004), and cholecystectomy (OR=2.56, *P*=1.05×10^-6^) on GERD. These findings were replicated in the FinnGen cohort and were also confirmed by BWMR and multivariable MR analyses. Additionally, mediation analyses demonstrated that cholelithiasis and cholecystitis acted as partial mediators, linking BMI causally to GERD. Conversely, GERD exhibited causal effect on cholelithiasis (OR=1.52, *P*=9.17×10^-30^) and cholecystitis (OR=1.90, *P*=3.32×10^-28^), which remained significant after BWMR and multivariable MR analyses. Mediation analyses further revealed significant mediating effect of GERD on how BMI influenced cholelithiasis/cholecystitis.

**Conclusion:**

Our study elucidates the bidirectional causal linkages involving cholelithiasis, cholecystitis, cholecystectomy, and GERD. These results highlight the significance of GERD risk assessment in individuals suffering from biliary diseases and vice versa.

## Introduction

Highlights

- Investigated the causal linkages between biliary disorders and gastroesophageal reflux disease (GERD) using MR method.
- Found evidence supporting the causal effects of cholelithiasis, cholecystitis, and cholecystectomy on increased risk of GERD.
- Explored bidirectional causality between GERD and biliary disorders, revealing GERD as a potential risk factor for cholelithiasis and cholecystitis.
- Utilized Bayesian weighted Mendelian randomization and multivariable Mendelian randomization to adjust for potential confounders, enhancing the robustness of causal inference.
- Identified cholelithiasis, cholecystitis, cholecystectomy (or GERD) as mediators in the causality between BMI and GERD (or cholelithiasis and cholecystitis), providing insights into disease mechanisms.

Biliary disorders and gastroesophageal reflux disease (GERD) are both prevalent digestive conditions, yet their causal connections remain uncertain. With advancements in scientific research, bile reflux is increasingly being recognized as an important pathogenic factor in GERD^1,2^. Although cholelithiasis, cholecystitis, and prior cholecystectomy are believed to contribute to bile reflux, it is still undetermined whether they definitively lead to GERD^3–5^. Additionally, some studies indicate that high co-occurrence rates of GERD with cholelithiasis and cholecystitis are due to common risk factors because their pathogenesis is closely related to surgery, and abnormalities in the vagus nerve and Cajal interstitial cells^4,6,7^.

Recently, Mendelian randomization (MR) has become widely used to clarify causal connections among complex risk factors and diseases^8^. MR addresses the causality involving exposure and outcome, which traditional experimental methods cannot effectively address because of confounding factors^8^. As of now, no MR study demonstrates the connections involving cholelithiasis, cholecystitis, cholecystectomy, and GERD.

Mediation analysis identifies intermediary factors that could serve as potential targets for interventions, thereby enhancing our understanding of disease causation^9^. The absence of robust causal assumptions in traditional nonindependent variable mediation analysis limits its applicability^9^. In this research, MR methods along with mediation analysis were conducted to examine whether biliary disorders (or GERD) served as mediators in the causality between BMI and GERD (or biliary disorders), given that BMI commonly contributes to the risk of GERD, cholelithiasis, and cholecystitis^10,11^.

This research explored the causal linkages involving cholelithiasis, cholecystitis, cholecystectomy, and GERD using genetic methods. Furthermore, MR mediation analyses were conducted to offer causal insights into the role of cholelithiasis, cholecystitis, and cholecystectomy (or GERD) in the development of GERD (or cholelithiasis and cholecystitis) and to offer new insights into early prevention.

## Methods

### Study design and data sources

**Figure 1:**
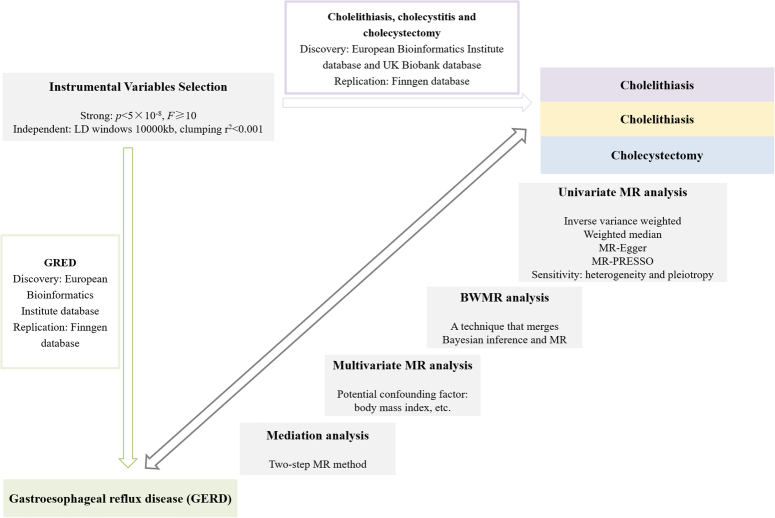
Study overview BWMR, Bayesian weighted MR; MR, Mendelian randomization.

To determine potential causal linkages involving cholelithiasis, cholecystitis, cholecystectomy, and GERD, we performed a MR study. MR study relys on three key principles: ensuring robust relationships of instrumental variables (IVs) with exposure, maintaining the exclusivity of IVs from potential outcome risk factors, and asserting the independence of IVs in influencing outcomes solely through exposure. Figure 1 depicted the methodological layout. Genome-wide association study (GWAS) data were extracted via the European Bioinformatics Institute (EBI) database, comprising 129 080, 26 122, and 9820 patients and 473 524, 461 431, and 461 431 controls for GERD, cholelithiasis, and cholecystitis, respectively. This database represents a comprehensive interdisciplinary data resource across continents and is accessible to those in the field of life sciences. Additionally, we extracted data from 18 319 patients who underwent cholecystectomy and 444 614 controls from the UK Biobank database. Furthermore, genetic data from the FinnGen database were acquired to perform external verification. Table 1 displayed the summary statistics of GWAS data. This research adhered to the strengthening the reporting of cohort, cross-sectional, and case–control studies in surgery (STROCSS) criteria^12^ (Supplemental Digital Content 1, http://links.lww.com/JS9/D206).

**Table 1:** Detailed information for the GWAS data.

Outcomes	ID	Sample size	Cases/Controls	PMID	Year	Population
GERD	ebi-a-GCST90000514	602604	129 080/473 524	34 187 846	2021	European
GERD	finngen_R9_K11_REFLUX	222545	32 232/190 313	NA	2023	European
Cholelithiasis	ebi-a-GCST90018819	487553	26 122/461 431	34 594 039	2021	European
Cholelithiasis	finngen_R9_K11_CHOLELITH	355443	44 582/310 861	NA	2023	European
Cholecystitis	ebi-a-GCST90018818	471251	9820/461 431	34 594 039	2021	European
Cholecystitis	finngen_R9_K11_CHOLECYST	45496	5237/40 259	NA	2023	European
Cholecystectomy	ukb-b-6235	462933	18 319/444 614	NA	2018	European
Cholecystectomy	finngen_R9_K11_CHOLECYSTECTOMY	208220	37 768/170 452	NA	2023	European

GERD, gastroesophageal reflux disease; GWAS, Genome-wide association study.

### Informed consent and ethical approval statement

Because individuals contributing to this MR study obtained necessary informed consent and ethical approvals from their respective studies, no additional informed consent or ethical approval was required.

### IVs selection

Single nucleotide polymorphisms (SNPs) serve as IVs, because random distribution of genetic mutations at conception lessens the impacts of confounding variables. For the genetic IVs, we selected SNPs meeting *P*<5×10^-8^. To mitigate linkage disequilibrium (LD) effect and reduce correlation level multiplicity, we applied LD clumping (LD windows 10 000 kb, clumping r^2^<0.001). Subsequently, weak IVs (F<10) were filtered out to ensure a strong IV-exposure correlation. Then, we harmonized SNPs for consistent effect estimates, omitting palindromic SNPs and those with incompatible effect allele frequencies.

### Univariable MR analysis

Causal estimation was performed using ‘TwoSampleMR’ package, and outliers were filtered out using ‘MR-PRESSO’ package. The analytical methods used included inverse variance weighting (IVW), MR-Egger, weighted median, and MR-PRESSO. As the most statistically efficient MR method, IVW served as the principal tool for MR analysis^13^. The MR-PRESSO outlier test identified potential outliers. Heterogeneity among the IVs was assessed using the Cochran *Q* test of the IVW method. Directional pleiotropy was evaluated via the intercept of MR-Egger regression. A leave-one-out analysis checked for SNP influence or bias on MR results. The findings were expressed as odds ratios (OR) and 95% CI. Statistical significance was defined as *P*<0.05.

### Bayesian weighted Mendelian randomization

Bayesian Weighted Mendelian Randomization (BWMR) was performed using ‘BWMR’ package. BWMR combines Bayesian inference and MR to address confounding factors and integrate prior knowledge with observed data^14^. This method addresses uncertainty in estimating weak effects and minor horizontal pleiotropy, while also detecting outliers caused by significant horizontal pleiotropy. By conducting extensive simulations and analyzing real-world data, it provides a more precise estimation of causal linkages, thus enhancing the reliability of our results.

### Multivariable MR analysis

Multivariable MR analysis was conducted via ‘MVMR’ package. Through integrating all exposures into a unified model, multivariable MR extends univariable MR in that it predicts the causal impacts of different risk variables on outcomes^15^. We conducted multivariable MR analyses to demonstrate that exposure was independent of potential confounding factors, considering that blood lipids, BMI, smoking status, alcohol consumption frequency, hypertension, and diabetes are potential risk factors for cholelithiasis, cholecystitis, cholecystectomy, and GERD. Significantly associated SNPs were extracted and combined with existing exposure to IVs. Following the exclusion of duplicate SNPs, we determined the effects and their respective standard deviations from exposure and outcome data. Causal relationships in the multivariable MR analyses were inferred using the IVW method, based on weighted linear regression.

### Mediation analysis

Mediation analyses were conducted to explore how cholelithiasis, cholecystitis, and cholecystectomy mediated the relationship between BMI and GERD, and how GERD mediated the relationships between BMI and cholelithiasis/cholecystitis. The method involved: (i) assessing total effects of BMI on these conditions; (ii) determining direct effect (a) of BMI on GERD (or cholelithiasis, cholecystitis, and cholecystectomy); (iii) evaluating direct effects (b) of cholelithiasis, cholecystitis, and cholecystectomy on GERD or vice versa. Finally, mediation effect was equal to a×b. And, the mediation effect divided by the total effect quantified how much of the relationship was mediated.

## Results

### Causal effects of biliary disorders on GERD

#### Univariable MR analysis

**Figure 2:**
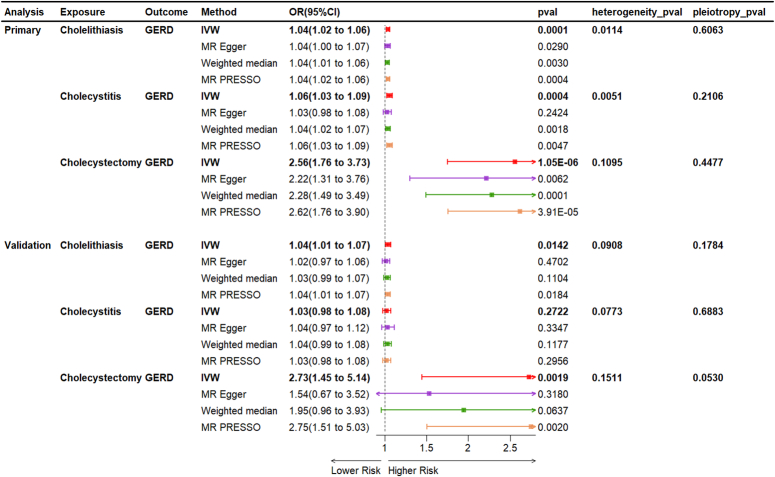
Univariable MR analysis of causal effects of biliary disorders on gastroesophageal reflux disease MR, Mendelian randomization.

As illustrated in Figure 2, cholelithiasis (OR=1.04, 95% CI=1.02–1.06, *P*=0.0001) and cholecystitis (OR=1.06, 95% CI=1.03–1.09, *P*=0.0004) were found to increase the incidence of GERD according to the univariable MR analyses using the IVW method. To our surprise, after removal of gallbladder, the risk of GERD increased by OR=2.56 (95% CI=1.76–3.73, *P*=1.05×10^-6^). Other models, such as MR-PRESSO, exhibited similar trends, reinforcing the robustness of these findings. Cochran *Q* test indicated heterogeneity regarding the effects of cholelithiasis (*P*=0.0114) and cholecystitis (*P*=0.0051) on GERD, possibly due to different analytical platforms, experiments, and populations; however, this did not compromise the robustness of our results, which was acceptable^16^. The Egger intercept results showed that horizontal pleiotropic effects were unlikely to bias the causal contribution of cholelithiasis, cholecystitis, and cholecystectomy to GERD (all *P*>0.05). Additionally, these results were not fundamentally impacted by the removal of any particular SNP, indicating the absence of outliers that would bias MR estimates, and thereby rendering the MR results reliable (Supplementary Fig. 1, Supplemental Digital Content 2, http://links.lww.com/JS9/D207).

To ensure the consistency of results across different datasets, we replicated the MR analyses in the FinnGen cohort using the same significant variant strategy. In the FinnGen cohort, the causal effects of cholelithiasis (OR=1.04, 95% CI: 1.01–1.07, *P*=0.0142) and cholecystectomy (OR=2.73, 95% CI: 1.45–5.14, *P*=0.0019) on GERD remained significant (Fig. 2). However, the causal impact of cholecystitis on GERD disappeared (OR=1.03, 95% CI: 0.98–1.08, *P*=0.2722).

### BWMR analysis

**Figure 3:**
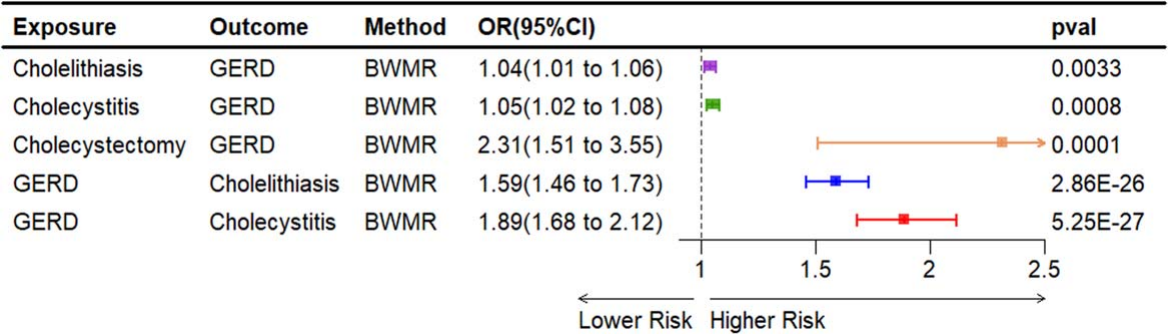
Bayesian weighted MR analysis MR, Mendelian randomization.

We used BWMR methods to validate our study, confirming that cholelithiasis (OR=1.04, 95% CI: 1.01–1.06, *P*=0.0033), cholecystitis (OR=1.05, 95% CI: 1.02–1.08, *P*=0.0008), and cholecystectomy (OR=2.31, 95% CI: 1.51–3.55, *P*=0.0001), were linked to a higher incidence of GERD (Fig. 3).

### Multivariable MR analysis

**Figure 4:**
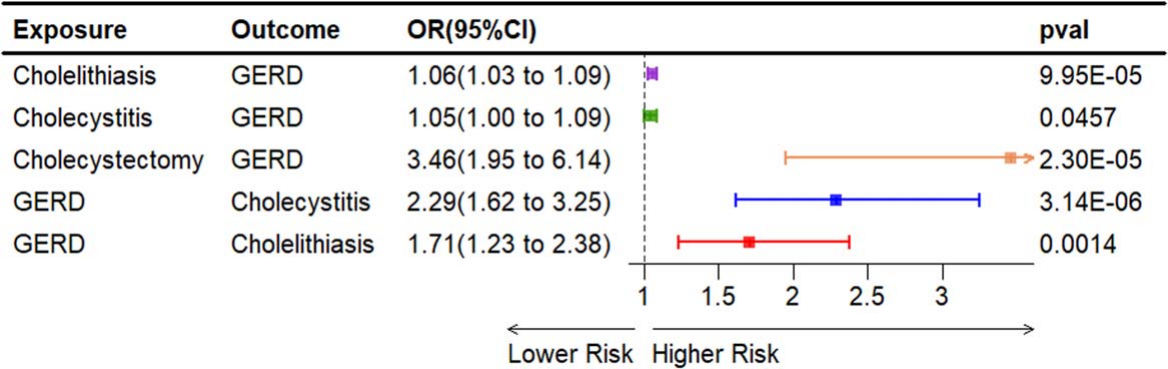
Multivariable analysis MR, Mendelian randomization.

After controlling for nine potential confounding variables (blood lipids, BMI, smoking status, alcohol consumption frequency, hypertension, and diabetes) in the multivariable MR analyses, the causal effects of cholelithiasis (OR=1.06, 95% CI: 1.03–1.09, *P*=9.95×10^-5^), cholecystitis (OR=1.05, 95% CI: 1.00–1.09, *P*=0.0457), and cholecystectomy (OR=3.46, 95% CI: 1.95–6.14, *P*=2.30×10^-5^) on GERD remained significant, and the direction of causal association was consistent with that of previous findings (Fig. 4).

### Mediating roles of biliary disorders on the causal linkage between BMI and GERD

Because BMI is a common etiological factor for cholelithiasis, cholecystitis, and GERD^10,11^, it was necessary to explore the mediating roles of biliary conditions on the linkage between BMI and GERD. As shown in Table 2, we observed that BMI increased the risk of GERD (β=0.814, *P*=2.79×10^-167^). Regarding direct effects, BMI was positively related to cholelithiasis (β=0.553, *P*=6.47×10^-61^), cholecystitis (β=0.663, *P*=8.50×10^-51^), and cholecystectomy (β=0.027, *P*=6.37×10^-61^) (direct effect a). Additionally, cholelithiasis (β=0.041, *P*=0.0001), cholecystitis (β=0.057, *P*=0.0004), and cholecystectomy (β=0.940, *P*=1.05×10^-6^) causally increased the risk of GERD (direct effect b). Thus, the proportion of mediation effects mediated by cholelithiasis, cholecystitis, and cholecystectomy for the linkage between BMI and GERD were 2.802% (95% CI: 1.345–4.259%), 4.608% (95% CI: 1.978–7.238%), and 3.123% (95% CI: 1.812–4.43%), respectively.

**Table 2:** Mediation MR analysis outcomes.

			Total effect			Direct effect a			Direct effect b			Mediation effect	
Exposure	Mediator	Outcome	β	SE	Pval	β	SE	Pval	β	SE	Pval	Effect size (95% CI)	Proportion % (95% CI)
BMI	Cholelithiasis	GERD	0.814	0.030	2.79E-167	0.553	0.034	6.47E-61	0.041	0.011	0.0001	0.023 (0.011–0.035)	2.802 (1.345–4.259)
BMI	Cholecystitis	GERD	0.814	0.030	2.79E-167	0.663	0.044	8.50E-51	0.057	0.016	0.0004	0.037 (0.016–0.06)	4.608 (1.978–7.238)
BMI	Cholecystectomy	GERD	0.814	0.030	2.79E-167	0.027	0.002	6.37E-61	0.940	0.193	1.05E-06	0.025 (0.014–0.040)	3.123 (1.812–4.430)
BMI	GERD	Cholelithiasis	0.553	0.034	6.47E-61	0.814	0.030	2.79E-167	0.417	0.037	9.17E-30	0.339 (0.276–0.402)	61.294 (49.831–72.756)
BMI	GERD	Cholecystitis	0.663	0.044	8.50E-51	0.814	0.030	2.79E-167	0.641	0.058	3.32E-28	0.521 (0.463–0.580)	78.669 (69.838–87.500)

GERD, gastroesophageal reflux disease

## Causal effect of GERD on biliary disorders

### Univariable MR analysis

**Figure 5:**
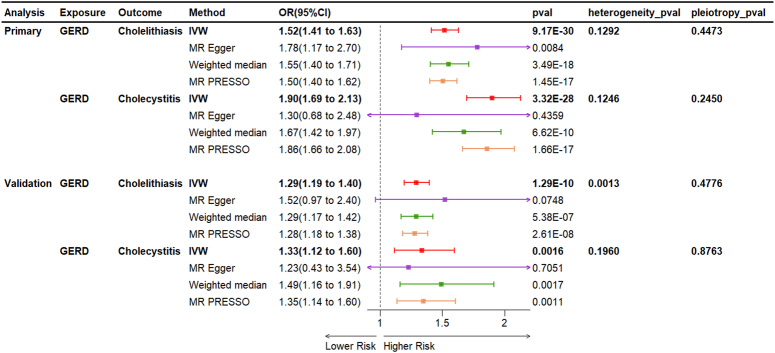
Univariable MR analysis of causal effect of gastroesophageal reflux disease on biliary disorders MR, Mendelian randomization.

As illustrated in Figure 5, ORs of the IVW methods were 1.52 (95% CI: 1.41–1.63, *P*=9.17×10^-30^), and 1.90 (95% CI: 1.69–2.13, *P*=3.32×10^-28^) for the risk of GERD on cholelithiasis, cholecystitis, and cholecystectomy, respectively. Other methods, such as MR-PRESSO approaches, could duplicate these findings (Fig. 5). According to sensitivity analyses, neither heterogeneity nor horizontal pleiotropy was identified (all *P*>0.05) (Fig. 5). The validity of the data was demonstrated by leave-one-out analyses, which revealed that no individual SNP influenced any given MR outcome (Supplementary Fig. 2, Supplemental Digital Content 3, http://links.lww.com/JS9/D208). In the FinnGen database, ORs of the IVW results were 1.29 (95% CI: 1.19–1.40, *P*=1.29×10^-10^), and 1.33 (95% CI: 1.12–1.60, *P*=0.0016) for the risk of GERD on cholelithiasis, and cholecystitis, respectively (Fig. 5).

### BWMR analysis

Validation with BWMR showed that ORs were 1.59 (95% CI: 1.46–1.73, *P*=2.86×10^-26^), and 1.89 (95% CI: 1.68–2.12, *P*=5.25×10^-27^) for the risk of GERD on cholelithiasis, and cholecystitis, respectively (Fig. 3).

### Multivariable MR analysis

Following adjustment for confounding variables such as blood lipids, BMI, smoking status, alcohol consumption frequency, hypertension, and diabetes, ORs were 2.29 (95% CI: 1.62–3.25, *P*=3.14×10^-6^), and 1.71 (95% CI: 1.23–2.38, *P*=0.0014) for the risk of GERD on cholelithiasis, and cholecystitis, respectively (Fig. 4).

### Mediating role of GERD on the causal linkages between BMI and biliary disorders

As shown in Table 2, BMI could increase the incidence of cholelithiasis (β=0.553, *P*=6.47×10^-61^) and cholecystitis (β=0.663, *P* =8.50×10^-51^). The direct effect a indicated that BMI also increased the incidence of GERD (β=0.814, *P*=2.79×10^-167^). Additionally, the direct effect b demonstrated causal linkages between GERD and cholelithiasis (β=0.417, *P*=9.17×10^-30^) and cholecystitis (β=0.641, *P*=3.32×10^-28^). Thus, the mediation proportion of GERD on the causal linkage between BMI and cholelithiasis was 61.294% (95% CI: 49.831–72.756%), and that on the causal linkage between BMI and cholecystitis was 78.669% (95% CI: 69.838–87.500%).

## Discussion

The complex linkages between cholelithiasis, cholecystitis, cholecystectomy, and GERD were investigated in our study using MR methods. Our results of univariable analyses demonstrated that cholelithiasis, cholecystitis, and cholecystectomy were linked to a higher incidence of GERD, which was consistently observed across various MR methods, indicating the high reliability of our findings. Additionally, validation analyses performed using the FinnGen database confirmed that cholelithiasis and cholecystectomy were positively causally associated with GERD, although the causal effect of cholecystitis on GERD was not significant in this cohort. This disparity underscores the necessity for replication studies across different populations to ensure the generalizability of the research findings. The evidence supporting causal linkages between cholelithiasis, cholecystitis, cholecystectomy and GERD was strengthened by BWMR and multivariable MR analyses. Moreover, cholelithiasis, cholecystitis, and cholecystectomy were found to partially mediate the causal linkage between BMI and GERD, suggesting potential mechanistic pathways linking these diseases. In order to determine if GERD contributed to the development of cholelithiasis and cholecystitis, our study went further into reverse causality. The findings showed strong causal linkages involving GERD and cholelithiasis/cholecystitis, supporting the bidirectional association between gastrointestinal and biliary diseases.

Previous observational studies examined the relationships between cholelithiasis, cholecystitis, cholecystectomy, and GERD. Gushcha *et al*.^17^ reported that cholecystitis often coexists with reflux esophagitis (20–60%). Additionally, a study observed that the risk of refractory GERD in individuals with cholecystitis was 6.776 (1.917–23.956) times higher than that in healthy controls^4^. Hepatobiliary scintigraphy has shown that 23% of individuals with acute and chronic cholecystitis suffered from duodenogastric reflux^18^, and prolonged bile reflux into the esophagus can erode the mucosal barrier, resulting in GERD^2^. In the West, individuals suffering from cholelithiasis or a history of cholecystectomy are more likely to experience duodenogastric reflux than healthy controls^19,20^. Several prospective studies have found that the incidence of GERD significantly increases following cholecystectomy^21,22^, although a case–control study indicated that neither gallstones nor cholecystectomy leads to GERD^23^. Currently, epidemiological evidence regarding the causal impacts of biliary conditions on GERD is inconsistent, perhaps due to interference from other uncontrollable variables such as hypertension, diabetes, smoking status, and alcohol consumption frequency. Using various MR methods, our investigation confirmed the causal impacts of biliary disorders on GERD. Additionally, biliary disorders played minor mediating roles in the causal impact of BMI on GERD.

Next, we attempted to explain the underlying reasons. In our opinion, an increased incidence of GERD associated with cholelithiasis, cholecystitis, and cholecystectomy may be attributed to the long-term pathological bile reflux into esophagus. Previous research has indicated that individuals with biliary illness are more vulnerable to duodenal-biliary reflux^24^, while patients with GERD not only had significant gastric acid-peptic reflux in the esophagus but also often had bile-gastric acid combined reflux^25,26^. The gallbladder serves as a reservoir and concentrator of bile, with ~20–25% of liver-produced bile entering intestine immediately. In conditions such as gallstones and cholecystitis, the volume of hepatic bile entering the duodenum increases owing to a decrease in gallbladder water absorption^27^. Similarly, following cholecystectomy, patients experience elevated bile duct pressure and excessive hepatic bile reflux into the duodenum, exceeding its clearance capacity^5^. Additionally, direct postoperative nerve damage between the gallbladder and duodenum can cause dysfunction of the Oddi sphincter^28^, making duodenogastric reflux more common. Prolonged reflux of bile acids into the esophagus disrupts the epithelial barrier, causing an influx of H^+^ ions and inactivation of sodium pumps on the cell membrane^29^. Refluxed bile also significantly reduces mucosal ATP levels, impairs sodium pump function^30^, and together causes intracellular water and sodium retention, leading to cell death due to cellular swelling. High concentrations of bile acids can directly damage cell membranes and tight junctions, dissolve mucosal cell lipid bilayers, and impair the esophageal mucosal antireflux barrier, leading to erosion, ulcers, and metaplasia, with pathology severity increasing with reflux severity^26,31^. Using MR methods, we revealed that the occurrence of cholelithiasis, cholecystitis, and cholecystectomy can lead to an increased incidence of GERD.

In this study, we also conducted reverse assessment to determine whether GERD was causally related to cholelithiasis and cholecystitis and found that GERD was a causative factor for both conditions. Following adjustment for confounding factors such as hypertension and diabetes, GERD exhibited an increased causal impact on cholelithiasis (OR increased from 1.52 to 2.29) and a decreased causal impact on cholecystitis (OR decreased from 1.90 to 1.71). Furthermore, GERD showed a considerable mediating effect on the causal pathways linking BMI to cholelithiasis and cholecystitis (61.294 and 78.669%, respectively).

According to previous research, individuals suffering from GERD are more vulnerable to cholelithiasis and cholecystitis^32,33^. This can be attributed to the following five factors: First, autonomic nervous dysfunction in the gastrointestinal tract is common in GERD^34^, which affects the co-ordination of gallbladder contraction and Oddi sphincter movement, leading to bile stasis, formation of biliary sludge, and ultimately the occurrence of cholelithiasis and cholecystitis. Second, delayed gastric emptying often accompanies GERD, reducing the effective stimulation of the duodenum by food, resulting in reduced secretion of cholecystokinin and abnormal gallbladder contractions, ultimately increasing the risk of biliary diseases. Third, proton pump inhibitor (PPI) is frequently prescribed to GERD patients. PPIs can slow down the emptying of the stomach and raise the concentration of bile in the stomach, which reduces the release of cholecystokinin^35^. Meanwhile, a recent study found that chronic PPI administration alters fecal microbiota composition in rats, and metabolic changes in the body are similar to those in rats on a high-fat diet, which can induce the formation of gallstones^36^. Fourth, long-term gastrointestinal disorders also exacerbate the generation of negative emotions, a phenomenon that is more pronounced in patients with GERD^37^, and psychiatric disorders have been shown to be linked to an elevated risk of cholelithiasis and cholecystitis^38^. Fifth, GERD is sometimes attributed to Helicobacter pylori infection^39^. Additionally, one research indicated a favorable association linking H. pylori to a higher incidence of cholelithiasis and cholecystitis^40^. Therefore, eradicating H. pylori infection presents a new perspective for treating GERD alone or for collectively addressing GERD, cholecystitis, and cholelithiasis^41–43^.

Our findings have several clinical implications. First, for patients with cholelithiasis or cholecystitis, especially those requiring cholecystectomy, the risk of GERD should be assessed. Preventive measures should be taken to mitigate GERD occurrence, such as advising patients to avoid excessive intake of irritant foods and to maintain a healthy lifestyle. Second, proactive treatment of GERD is needed to prevent cholelithiasis and cholecystitis. For individuals suffering from GERD, especially for obese patients, there is a possibility of concurrent biliary diseases. Therefore, it is important to treat both GERD and biliary conditions simultaneously to enhance therapeutic outcomes.

This study was based on MR methods, which greatly minimized the bias caused by confounding factors. Second, to obtain credible causal inferences and ensure consistency of the results, we extracted GWAS data from several large databases for causal estimation. Additionally, we used multiple MR methods to reinforce the robustness of our findings. In the present study, no evidence of pleiotropy was found in any of the analyses, indicating that the causal linkages between cholelithiasis, cholecystitis, cholecystectomy and GERD were not due to confounding factors.

This investigation had several constraints. Initially, the publicly available GWAS data limited the exploration of subgroups, such as stratification by age and sex. Secondly, we were unable to distinguish causality based on disease severity because, to our knowledge, no SNPs have been identified as severity-related for cholelithiasis, cholecystitis, cholecystectomy, and GERD. Finally, GWAS data were derived from the Finnish, British, and European populations. Additional study is required to confirm whether the findings of this study are applicable to other regions such as Asia and Africa.

## Conclusion

Using MR methods, this study demonstrated bidirectional causal linkages between GERD and biliary disorders. These causal effects persisted independently of potential confounding factors such as BMI. Additionally, cholelithiasis, cholecystitis, and cholecystectomy serve as minor mediators in the causality between BMI and GERD, whereas GERD serves as a significant mediator in the causalities between BMI and cholelithiasis/cholecystitis. This research enhances comprehension of the relationships between cholelithiasis, cholecystitis, cholecystectomy, and GERD. Patients with biliary diseases should be closely monitored for the possibility of developing GERD and for early intervention, and vice versa.

## Ethical approval

Our research utilized publicly available GWAS data, which have obtained ethical approval from involving patients. As such, additional ethical approval was not required for this analysis.

## Consent

Due to the fact that the research data are sourced from publicly available GWAS databases, they have already obtained appropriate ethical approval and reference numbers in the original studies. Therefore, no additional ethical approval is required for the research presented in our paper.

## Source of funding

This work is supported by the 1•3•5 project for disciplines of excellence–Clinical Research Incubation Project, West China Hospital, Sichuan University (2018HXFH020), Regional Innovation and Collaboration projects of Sichuan Provincial Department of Science and Technology (2021YFQ0026), National Natural Science Foundation Regional Innovation and Development (U20A20394), National Nature Science Foundation of China (82000514), key projects of Sichuan Provincial Department of Science and Technology (2021YFS0222) and China Postdoctoral Science Foundation (2020M673241).

## Author contribution

H.L.: conceptualization, formal analysis, methodology, software, validation, visualization, and writing – original draft; R.L.: formal analysis, methodology, software, validation, visualization, and writing – original draft; Q.S.: methodology, supervision, validation, and writing – review and editing; Y.G.: methodology, supervision, and writing – review and editing; Y.L.: software; Y.Y.: conceptualization, funding acquisition, investigation, methodology, resources, and writing – review and editing; L.C.: conceptualization, funding acquisition, supervision, resources, and writing – review and editing.

## Conflicts of interest disclosure

The authors declare no conflicts of interest.

## Research registration unique identifying number (UIN)

Our research utilized publicly available GWAS data and the GWAS ID can be obtained from the article. (ebi-a-GCST90000514, finngen_R9_K11_REFLUX, ebi-a-GCST90018819, finngen_R9_K11_CHOLELITH, ebi-a-GCST90018818, finngen_R9_K11_CHOLECYST, ukb-b-6235, finngen_R9_K11_CHOLECYSTECTOMY).

## Guarantor

Longqi Chen.

## Date availability statement

Publicly available datasets were analyzed in this study. These datasets can be found at the following URLs: IEU OpenGWAS project (https://gwas.mrcieu.ac.uk/) and FinnGen (https://r9.risteys.finngen.fi/). The data used in this study were obtained from publicly available GWAS databases and accessed openly.

## Provenance and peer review

Not commissioned, externally peer-reviewed.

## Supplementary Material

**Figure:**
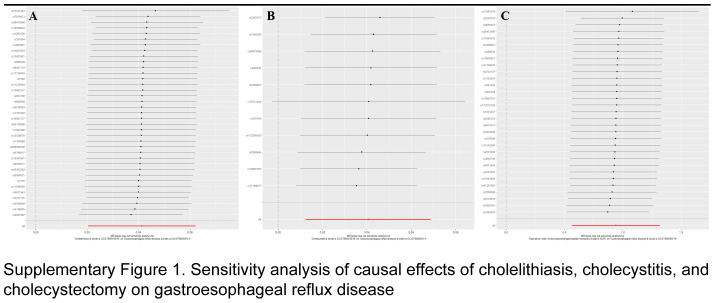


**Figure:**